# Medication Errors and Gaps in Medication Discharge Planning for Hospitalized Older Adults: A Prospective Cohort Study

**DOI:** 10.1007/s11606-025-09973-x

**Published:** 2025-11-19

**Authors:** Timothy S. Anderson, Linnea M. Wilson, Brianna X. Wang, Michael A. Steinman, Mara A. Schonberg, Edward R. Marcantonio, Shoshana J. Herzig

**Affiliations:** 1 Division of General Internal Medicine, University of Pittsburgh, Pittsburgh, PA USA; 2 Center for Healthcare Evaluation Research and Promotion (CHERP), VA Pittsburgh Healthcare System, Pittsburgh, PA USA; 3 Division of General Medicine, Beth Israel Deaconess Medical Center, Boston, MA USA; 4 Division of Geriatrics, University of California, San Francisco, San Francisco, CA USA; 5 Geriatrics and Extended Care, San Francisco VA Health Care System, San Francisco, CA USA; 6 Harvard Medical School, Boston, MA USA

**Keywords:** hospitalization, transitions of care, medication safety, older adults

## Abstract

**Background:**

Hospitalized older adults are commonly discharged with changes to antihypertensive and glucose-lowering (cardiometabolic) medications. Though adverse drug events remain a leading cause of readmissions, there is little contemporary data on how medication discharge planning is communicated and how often medication errors occur post-discharge.

**Objective:**

To assess older adults’ post-hospital medication use and ambulatory follow-up after receiving cardiometabolic medication changes during hospitalization.

**Design:**

Prospective cohort study from 11/2022 to 01/2024.

**Participants:**

Adults aged 65 years or older from discharged home from an academic medical center with changes to pre-admission cardiometabolic medications.

**Main Measures:**

Participants completed 7- and 90-day telephonic surveys on health status, medication use, and discharge planning. Self-report of medication use was compared to discharge summaries to identify medication errors (not initiating, not stopping, or taking incorrect dose). Multivariable regression models were used to identify characteristics associated with errors.

**Key Results:**

The cohort included 151 participants (median [IQR] age 74 [70–78] years; 54% male; 17% Black, 82% White, 41% frail). Participants were admitted with a median (IQR) of 3 (2–4) cardiometabolic medications and discharged with a median (IQR) of 2 (1–4) medication changes. Of the 319 individual medications changed at discharge, 33% were further modified by 90 days. Participants reported comprehensive medication discharge planning for only 13% of medication changes. Though 93% of participants reported they understood the purpose of each of their medications at discharge, 39% had ≥ 1 medication errors at 7 days and 50% at 90 days. Use of ≥ 5 cardiometabolic medications was associated with higher rates of medication errors at 7 days (IRR 1.63; 95% CI 1.07–2.48) and 90 days (IRR 1.66; 95% CI 1.13–2.45).

**Conclusions:**

Most hospitalized older adults discharged with cardiometabolic medication changes experienced medication errors or gaps in discharge planning. Steps to ensure all patients receive high-quality medication discharge planning are needed.

**Supplementary Information:**

The online version contains supplementary material available at 10.1007/s11606-025-09973-x.

## INTRODUCTION

One in six older adults experience a hospitalization annually.^1^ Over two-thirds of the older adult population have multiple chronic conditions of which the most common comorbidities are cardiometabolic conditions such as hypertension and diabetes.^2^ Hospitalized older adults are often discharged with changes to their chronic medications, with studies observing 10 to 16% of older adults’ glucose-lowering or antihypertensive medication regimens being intensified at hospital discharge.^3–7^

Adverse drug events remain a major driver of preventable rehospitalization in older adults.^8^ To reduce adverse drug events ensuring high-quality communication of medication changes and post-discharge plans to monitor medication changes is paramount.^8^ Though achieving accurate written medication reconciliation has become a major focus of hospital discharge efforts to reduce adverse drug events,^9–11^ less attention has been paid to how medication changes are enacted by patients. The time clinicians typically spend with patients on education at discharge is often limited to a matter of minutes^12^ and patient understanding of post-discharge care plans is often poor.^13^ However, few studies of discharge transitions have focused on medication use, and those that have largely predate the electronic health record (EHR) era, which has improved medication reconciliation and may have improved discharge understanding.^14^

Thus, we aimed to prospectively assess older adults’ adherence to antihypertensive and glucose-lowering medication changes made during hospitalization, receipt of medication discharge planning, and subsequent medication use and medication errors post-discharge.

## METHODS

### Study Cohort

This was a prospective cohort study of patients 65 years or older who were discharged home following acute hospitalization at a Boston-area tertiary academic medical center between November 2022 and January 2024. Patients were eligible if they were discharged with changes to home antihypertensive or glucose-lowering (hereafter, cardiometabolic) medications (eTable 1). All types of medication changes were eligible including new starts, dose changes, stops, and holds. Enrolled patients completed two phone surveys at 7 days and 90 days post-discharge. We focused on 7 days to capture immediate post-discharge medication use and 90 days to capture subsequent medication changes over an entire clinical episode as defined by Medicare.^15^ The Beth Israel Deaconess Medical Center Institutional Review Board approved the study. Verbal informed consent was obtained from all participants.

The study planned to enroll 150 participants. To assess eligibility, research staff reviewed discharge summaries for patients from three services which frequently prescribe changes to cardiometabolic medications (cardiology, medicine, and neurology) to identify those with cardiometabolic medication changes. Eligibility criteria included receiving primary care in the same health system, a primary language of English, not enrolled in hospice care, and not identified by the discharging clinician as having altered mental status at discharge. Eligible patients were mailed study information flyers and then contacted by telephone between 5 and 9 days after discharge. Upon enrollment, additional eligibility criteria were assessed. Participants who reported not managing their own medications were ineligible if the responsible caregiver was unable to participate in the survey with them, as were participants who scored 9 or greater, or 5–8 without a caregiver to assist, on the Short Blessed Test (Short Orientation-Memory-Concentration Test).^16^

### Survey Data Collection

Participants completed a structured telephone survey with research staff (hereafter 7-day survey) that included questions on health status, hospital care, health since returning home, and medication use (eTable 2). Health status was assessed using the FRAIL Scale,^17,18^ the Walter Index for 1-year mortality in older adults after hospitalization,^19^ and the Katz Index of Independence in Activities of Daily Living.^20^ Hospital care quality was assessed using the 3-Item Care Transitions Measure (CTM-3).^21^ Health after returning home was assessed by asking about common symptoms associated with blood pressure and diabetes treatment as well as emergency department (ED) visits and hospital readmissions.

Medication use was assessed by asking the participant to identify all antihypertensive and glucose-lowering medications they were taking, the current dose, whether the medication was changed at hospital discharge, and, if so, the previous dose and reason for change. If a participant did not mention a medication identified by chart review, the interviewer prompted them with the medication name. For medications that participants identified as changed, participants were asked if they were (a) recommended to complete outpatient primary care or specialty follow-up regarding their blood pressure or blood sugar, (b) instructed to monitor their response to the change through home blood pressure or blood glucose monitoring, and (c) informed of potential side effects.

At 90 days following discharge, participants were again surveyed on their cardiometabolic medication use and post-discharge health status (eTable 3). At 90 days, research assistants additionally abstracted data on cardiometabolic medication use through structured chart review.

### Medication Changes

First, discharge medication changes were assessed by comparing medications used at admission to two sources: discharge summaries and medication use reported by participants in the 7-day survey. Similarly, medication changes at 90 days were assessed by comparing medications reported in the 7-day survey to two sources: medications intended to be used based on chart review of primary care and specialist and medications reported by participants in the 90-day survey.

Medication changes were assessed at the medication class level and defined as started, stopped, held, dose increased, dose decreased, or a change in frequency. Medication regimen changes were then assessed at the patient level separately for antihypertensive regimens and glucose-lowering regimens. Regimens were categorized as unchanged, increased (at least one new medication added or dose increased), decreased (at least one medication stopped or dose decreased), substitutions (number of medications started or increased equal to the number of medications stopped or decreased), or temporary holds.

### Medication Errors

For each cardiometabolic medication, we identified medication errors by comparing patient-reported use at 7 days to the hospital discharge summary (used as the criterion standard), accounting for any changes between discharge and 7 days documented in the EHR (e.g., outpatient clinician directed a medication change in response to laboratory results). We defined medication errors as medications intended to be taken at discharge which were not used, medications intended to be stopped at discharge being taken, and medications being taken at a different dose than intended. We then identified additional medication errors which occurred between 7 and 90 days by comparing patient-reported 90-day use to chart review of the EHR. This 7-to-90-day post-discharge period was chosen to investigate ongoing medication challenges patients may face in the post-hospitalization period, beyond those directly related to hospital discharge.

### Medication Discharge Planning

For each patient-reported medication change, we measured the adequacy of medication discharge planning by assessing three domains: (1) whether a follow-up primary care or specialist visit to follow the medication changes was recommended, (2) whether home monitoring (home blood pressure or blood sugar monitoring) was recommended, and (3) whether side effects of the medication change were discussed at discharge. Using chart review we then identified whether outpatient primary care or specialist follow-up was recommended in the discharge instructions, the recommended timeframe, and actual completion of follow-up within 90 days.

### Post-Discharge Clinical Outcomes

We assessed symptoms commonly related to antihypertensive and glucose-lowering medications at 7 and 90 days. Symptoms included falls, syncope, unsteadiness (composite of reported falls, syncope, dizziness, orthostatic dizziness, or feeling unsteady when standing or walking), and hypoglycemic episodes. We similarly assessed ED visits and hospital readmissions through patient self-report confirmed by chart review.

### Statistical Analysis

We compared demographics and hospitalization characteristics between participants and eligible non-participants using standardized mean differences. Among participants, we used descriptive statistics to report measures of baseline health status, medication changes, and follow-up of medication changes. We constructed a Sankey diagram to describe medication changes from pre-admission to hospital discharge to 90 days post-discharge.

We constructed Poisson regression models to identify factors associated with experiencing one or more medication errors at 7 days and at 90 days post-discharge. First, univariate models were constructed separately examining age, sex, race, ethnicity, discharge disposition, discharge service, length of stay, primary care practice type, cardiometabolic medication polypharmacy (≥ 5 medications), number of cardiometabolic medication changes, CTM item 3 response, FRAIL Scale, Katz ADL Index, and the Walter Mortality Index. Second, multivariate Poisson regression models were constructed including the number of cardiometabolic medication changes and the covariates significantly associated with medication error outcomes in the univariate models. Robust standardized errors were used to allow estimation of incidence rate ratios.^22^ Analyses were conducted using SAS (v9.4) with a two-sided significance of *α* = 0.05.

## RESULTS

Of the 657 potentially eligible patients, 57 were ineligible, and 168 of the remaining 600 consented to the study (28.0%). Of these, 3 withdrew before initial survey completion and 14 had incomplete medication records (eFigure 1). The final cohort included 151 participants (median [IQR] age 74 [70–78] years, 54% male, 17% Black, 82% White) who completed the 7-day survey. Of these, 137 (90.7%) completed the 90-day survey, 4 died prior to 90 days, 5 withdrew, and 5 were lost to follow-up. Most participants had moderate (45.7%) or high (38.4%) 1-year risk of mortality and 41.1% were frail (Table 1). Compared to individuals who declined enrollment, participants were younger and less likely to be discharged with home health and had a shorter mean length of stay (eTable 4).

**Table 1:** Cohort Characteristics

Characteristic	Participants, no. (%)
Demographics	
Age, years, median (IQR)	74.0 (70.0–78.0)
Female sex	69 (45.7)
Race	
Black	26 (17.2)
White	124 (82.1)
Other	1 (0.7)
Ethnicity	
Hispanic or Latino	2 (1.3)
Not Hispanic or Latino	146 (96.7)
Unknown/refused to answer	3 (2.0)
Hospitalization characteristics	
Discharge disposition	
Home	109 (72.2)
Home with services	42 (27.8)
Discharge service	
Cardiology	84 (55.6)
Medicine	65 (43.0)
Neurology	2 (1.3)
Length of stay, days, median (IQR)	3.0 (2.0–6.0)
Primary care practice group^*^	
Academic	62 (41.1)
Community	82 (54.3)
Community health center	7 (4.6)
No. days survey was administered post-discharge, mean (SD)	7.0 (1.3)
Admission characteristics	
Admission medications	
No. cardiometabolic medications, median (IQR)	3 (2–4)
Any hypertension medication	140 (92.7)
Any diabetes medication	67 (44.4)
Admission diagnosis^†^	
Circulatory	80 (53.0)
Digestive	12 (7.9)
Endocrine	10 (6.6)
Abnormal findings	9 (6.0)
Genitourinary	8 (5.3)
Infectious disease	8 (5.3)
Neoplasms	7 (4.6)
Injury/poisoning	6 (4.0)
Respiratory	6 (4.0)
Other	5 (3.3)
Self-reported health status characteristics	
Chronic conditions	
Hypertension	121 (80.1)
Arthritis	92 (60.9)
Diabetes	66 (43.7)
Angina	49 (32.5)
Congestive heart failure	41 (27.2)
Kidney disease	39 (25.8)
Cancer (non-minor)	37 (24.5)
Heart attack	37 (24.5)
Asthma	27 (17.9)
Stroke	24 (15.9)
Chronic lung disease	18 (11.9)
FRAIL Scale	
Robust	24 (15.9)
Pre-frail	65 (43.0)
Frail	62 (41.1)
Katz Index of independence in activities of daily living^‡^	
Any deficit	36 (23.8)
Independent	115 (76.2)
Walter Index of 1-year risk of mortality	
Moderate	69 (45.7)
High	58 (38.4)
Very high	24 (15.9)

^*^All primary care practices were affiliated with the same health system. ^†^Circulatory diagnoses include coronary artery disease (*n* = 28), conduction (*n* = 16), heart failure (*n* = 15), valvular disease (*n* = 10), pericardial disease (*n* = 3), hypertension (*n* = 2), chest pain (*n* = 2), pulmonary embolism (*n* = 1), cerebral infarction (*n* = 1), hypotension (*n* = 1), and other/ill-defined cerebrovascular disease (*n* = 1). Endocrine diagnoses include diabetes mellitus with complication (*n* = 6) and fluid and electrolyte disorders (*n* = 4). Other diagnoses include blood/blood-forming organs, musculoskeletal system, nervous system, and skin/subcutaneous tissue. ^‡^Twenty-five (16.6%) of participants needed assistance with bathing, 22 (14.6%) with dressing, 12 (7.9%) with transferring, 7 (4.6%) with toileting, and 6 (4.0%) with eating. Data was not collected on continence independence

Nearly all (92.7%) participants agreed that they clearly understood the purpose of taking each of their medications at hospital discharge.

### Overall Medication Changes at Hospital Discharge

**Figure. 1:**
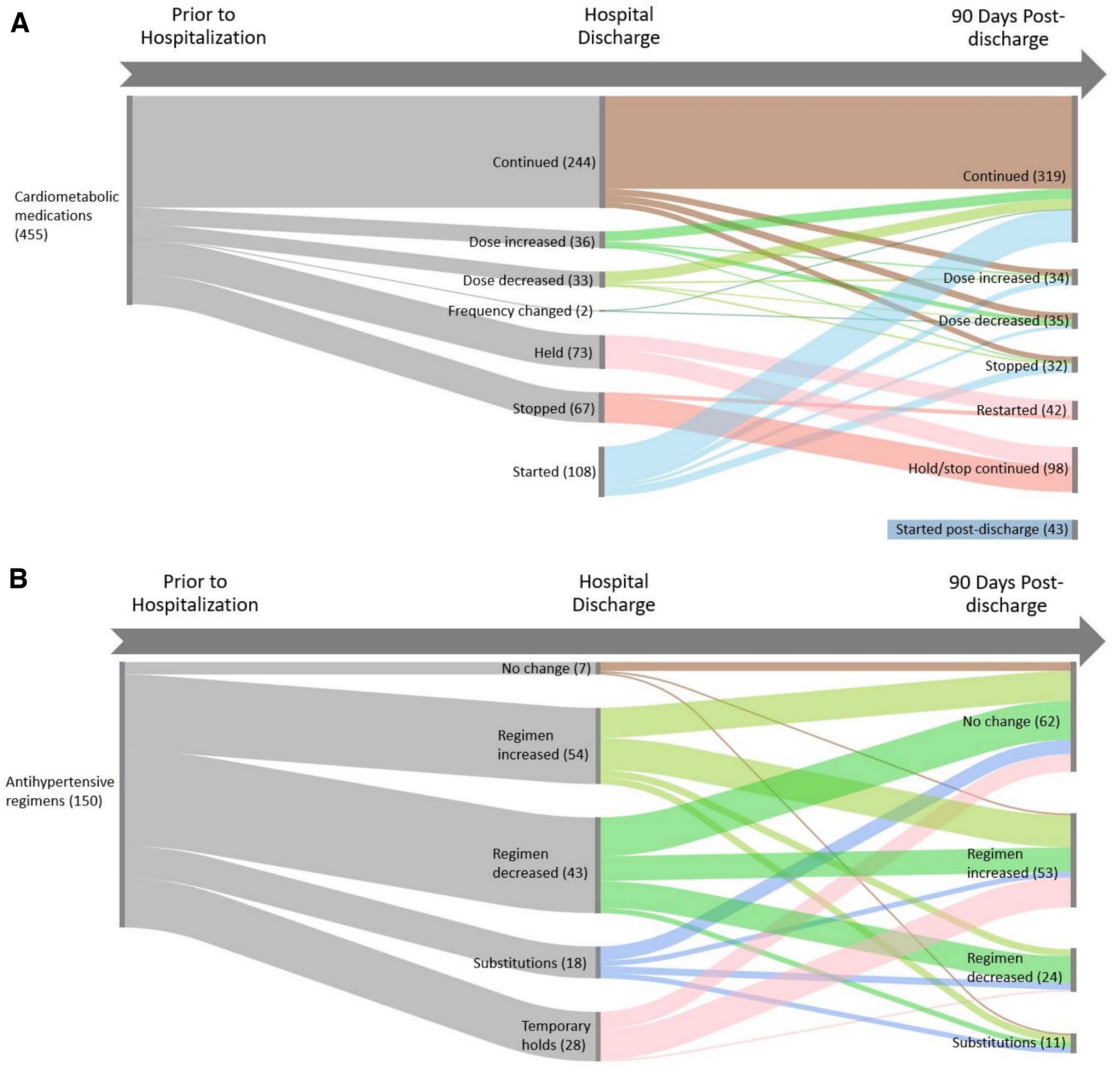

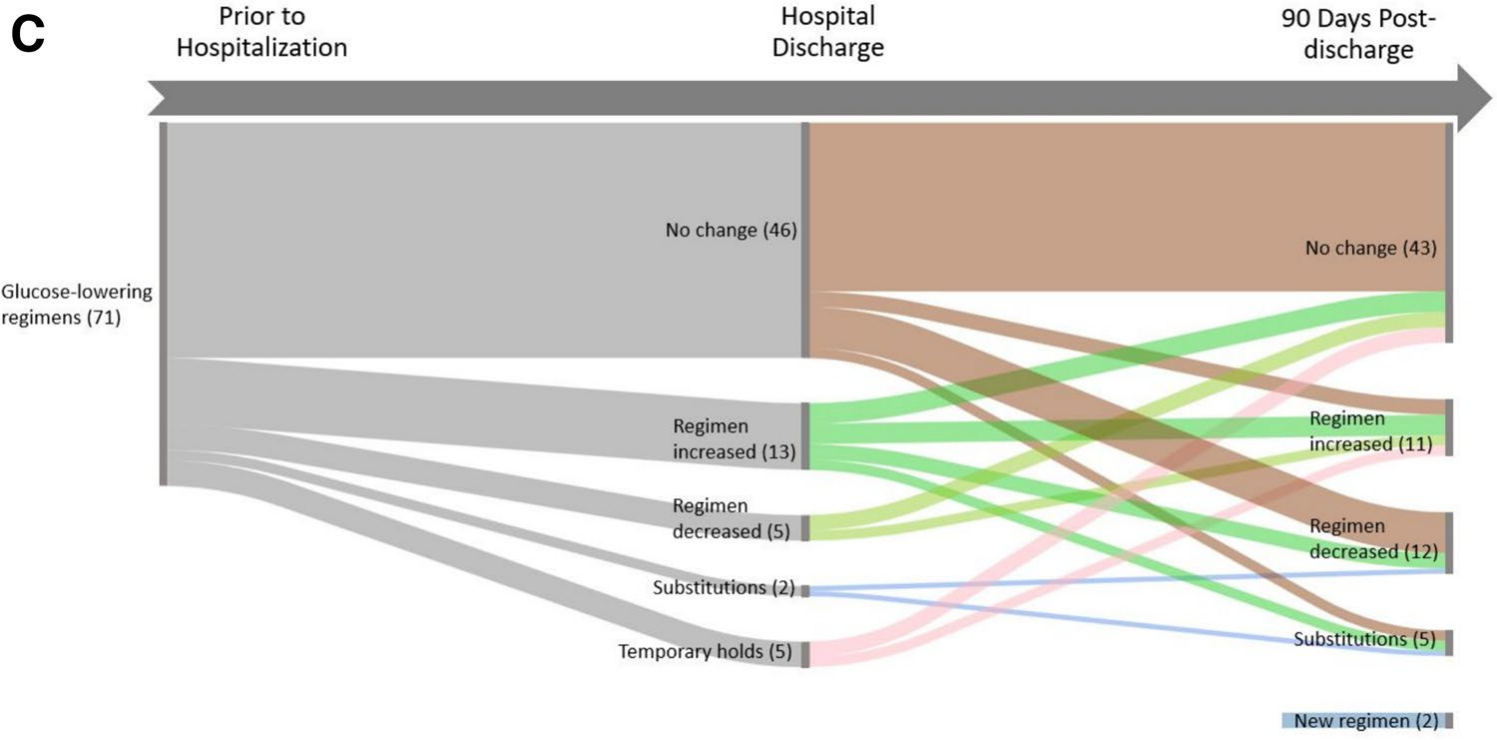
Changes to cardiometabolic medication use following acute hospitalization. **A** Individual cardiometabolic medication changes. **B** Participant-level antihypertensive regimen changes. **C** Participant-level glucose-lowering regimen changes.

On admission, the 151 participants were taking a median (IQR) of 3 (2–4) cardiometabolic medications, with 140 (93%) using antihypertensives and 67 (44%) using glucose-lowering medications. Participants were discharged with a median (IQR) of 2 (1–4) cardiometabolic medication changes (Fig. 1A). At 90 days after discharge, most participants (105/151; 69%) had additional cardiometabolic changes made.

Of the 319 cardiometabolic medication changes made at discharge, 34% were initiations of new medications, 23% were medication holds, 22% were dose changes, and 21% were medication stops (eTable 5). At 90 days, 33% (105/319) of individual medication changes made at discharge were subsequently modified. Of the 71 medications with a dose change at discharge, 10% were increased and 25% were reduced or stopped. Of the 73 medications held at discharge, 83% were restarted. Of the 67 medications stopped at discharge, 16% were restarted. Of the 108 medications newly started at discharge, 17% were stopped. Additionally, 43 new cardiometabolic medications were started between discharge and 90 days.

### Antihypertensive Medication Changes

At discharge, 150 participants were using antihypertensives: 37% were discharged with increased regimens (including 10 participants newly started on antihypertensives), 29% with decreased regimens, 18% with temporary holds, 11% with substitutions, and 5% without changes (Fig. 1B). At 90 days, of the 150 participants with antihypertensive regimens, 35% were increased, 16% were decreased, 7% were substituted, and 41% had no changes.

### Glucose-Lowering Medication Changes

Of the 71 participants using glucose-lowering medications at discharge 18% were discharged with increased regimens, 7% with temporary holds, 5% with decreased regimens, 3% with substitutions, and 65% without changes (Fig. 1C). At 90 days, of the 71 participants with glucose-lowering regimens upon admission, 15% were increased, 17% were decreased, 7% were substituted, and 61% had no changes.

### Medication Errors at 7 Days

Of the 151 participants, 59 (39%) were identified as having medication errors at the 7-day follow-up interview; 36 had a single error and 23 had multiple. In adjusted analyses, male sex (IRR 1.99; 95% CI 1.03–3.15), having five or more cardiometabolic medications (IRR 1.63; 95% CI 1.07–2.48), and taking both antihypertensive and glucose-lowering medications (IRR 1.66; 95% CI 1.06–2.60) were associated with increased incidence of medication errors (Table 2).

**Table 2:** Factors Associated with Medication Errors at 7 Days Post-discharge

Characteristic	No. with error/total no. (%)	Unadjusted IRR (95% CI)	Adjusted IRR (95% CI)
Overall	59/151 (39.1)	-	-
Demographics			
Age, years			
< 75	40/88 (45.5)	Ref	-
≥ 75	19/63 (30.2)	0.66 (0.43 to 1.03)	-
Sex			
Female	16/69 (23.2)	Ref	Ref
Male	43/82 (52.4)	**2.26 (1.40 to 3.64)**	**1.99 (1.26 to 3.15)**
Race and ethnicity			
Non-Hispanic White	50/124 (40.3)	Ref	**-**
Other	9/27 (33.3)	0.83 (0.47 to 1.47)	**-**
Hospitalization characteristics			
Discharge disposition			
Home	43/109 (39.5)	Ref	-
Home with services	16/42 (38.1)	0.97 (0.62 to 1.51)	-
Discharge service			
Medicine	23/65 (35.4)	Ref	-
Cardiology/neurology	36/86 (41.9)	1.18 (0.78 to 1.79)	-
Length of stay, days	-	0.99 (0.94 to 1.03)	-
Primary care practice type			
Academic	27/62 (43.6)	Ref	-
Community/community health center	32/89 (36.0)	0.83 (0.56 to 1.23)	-
No. of cardiometabolic medications			
< 5	29/105 (27.6)	Ref	Ref
≥ 5	30/46 (65.2)	**2.36 (1.62 to 3.43)**	**1.63 (1.07 to 2.48)**
No. of cardiometabolic medication changes			
1	22/64 (34.4)	Ref	Ref
≥ 2	37/87 (42.5)	1.24 (0.81 to 1.88)	1.00 (0.65 to 1.53)
Cardiometabolic medication classes			
Single	20/81 (24.7)	Ref	Ref
Both	39/70 (55.7)	**2.26 (1.46 to 3.48)**	**1.66 (1.06 to 2.60)**
Clearly understood purpose of medications at discharge*			
Strongly agree/agree	55/140 (39.3)	Ref	-
Strongly disagree/disagree	4/11 (36.4)	0.93 (0.41 to 2.08)	-
FRAIL Scale			
Not frail	38/89 (42.7)	Ref	-
Frail	21/62 (33.9)	0.79 (0.52 to 1.21)	**-**
Activities of daily living			
No assistance needed	40/115 (34.8)	Ref	Ref
Any assistance needed	19/36 (52.8)	**1.52 (1.02 to 2.26)**	1.40 (0.99 to 1.99)
1-year risk of mortality			
Moderate	22/69 (31.9)	Ref	-
High	37/82 (45.1)	1.42 (0.93 to 2.15)	-

*IRR*, incident rate ratio. Bolded estimates are statistically significant at alpha = 0.05. ^*^Assessed from question 3 of the 3-Item Care Transitions Measure “When I left the hospital, I clearly understood the purpose for taking each of my medications”

### Medication Errors at 90 Days

Of the 137 participants completing the 90-day follow-up, 69 (50%) were identified as having additional medication errors between 7 and 90 days; 32 had a single error and 37 had multiple. Similar to the 7-day analyses, having five or more cardiometabolic medications (IRR 1.66; 95% CI 1.13–2.45) was associated with an increased incidence of medication errors at 90 days (eTable 6).

### Medication Discharge Planning and Follow-Up

**Figure 2.**
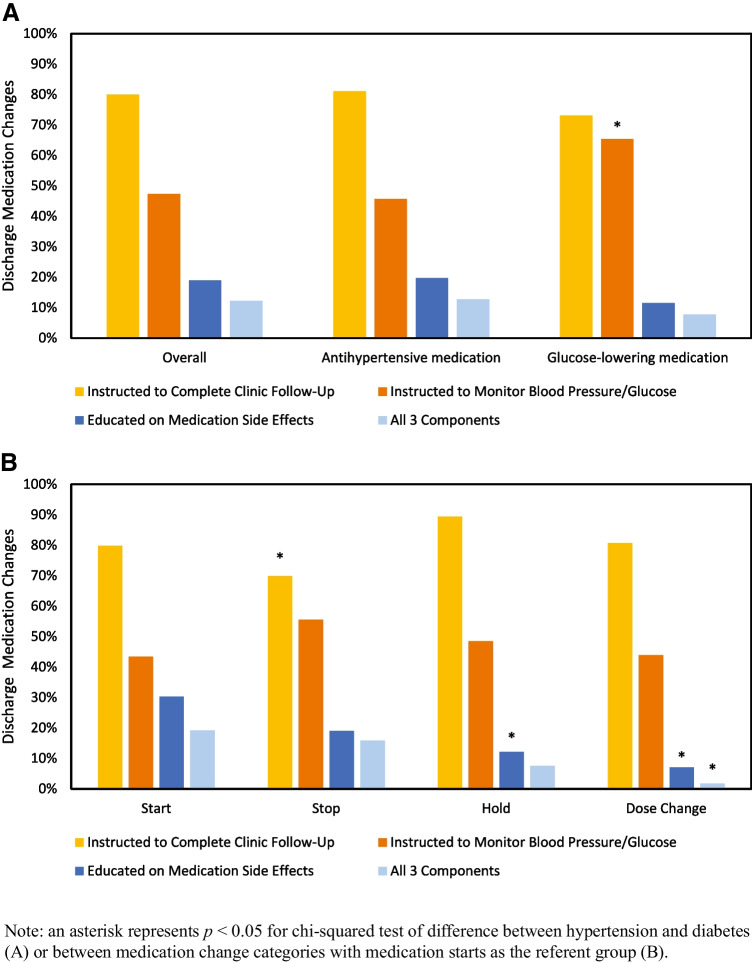
Receipt of discharge planning for medication changes. **A** Overall and by cardiometabolic medication group. **B** By medication change type.

Participants were asked about medication discharge planning for 275 of the 319 (86%) intended cardiometabolic medication changes at discharge that were correctly identified as having been changed. Participants reported being instructed to complete clinic follow-up for 80% of medication changes, to monitor home blood pressure or blood glucose for 47%, being educated about side effects for 19%, and receiving all three components of discharge medication planning for only 13% (Fig. 2). Receipt of all three components was less common for dose changes than for newly started medications (2% vs. 19%, *p* = 0.02).

Of the 151 participants, 9% did not receive a primary care follow-up visit and 28% did not receive a specialty care follow-up visit within 90 days of discharge (eFigure 2). Of the 138 participants with recommended primary care follow-up in the discharge summary, 99 (72.3%) received follow-up as directed, 27 (19.7%) received follow-up after the recommended interval, and 12 (8.8%) did not receive follow-up within 90 days. Of the 122 participants recommended specialist follow-up by the discharging team, 88 (72.1%) received follow-up as directed, 18 (14.8%) received follow-up after the recommended interval, and 16 (13.1%) did not receive follow-up within 90 days.

### Post-discharge Clinical Outcomes

Between discharge and the 7-day survey, 4 participants experienced falls, 6 experienced syncope, 13 experienced hypoglycemia, 74 experienced unsteadiness, and 4 had an ED visit or rehospitalization. Between 7 and 90 days post-discharge, 17 participants experienced falls, 5 experienced syncope, 18 experienced hypoglycemia, 80 experienced unsteadiness, 30 had an ED visit or rehospitalization, and 4 patients died or transitioned to hospice (Table 3).

**Table 3:** Adverse Events Following Discharge with Cardiometabolic Medication Changes

	**No.**	**No. (%) experiencing adverse event**					
		**Syncope**	**Falls**	Unsteadiness*	**Hypoglycemia**	**Hospital or ED visit**	**Any adverse event**
Discharge to 7 days							
Overall	151	6 (4.0)	4 (2.6)	74 (49.0)	13 (8.6)	4 (2.6)	77 (51.0)
Antihypertensive regimen	150	6 (4.0)	4 (2.7)	73 (48.7)	13 (8.7)	4 (2.7)	76 (50.7)
Diabetes regimen	71	4 (5.6)	3 (4.2)	41 (57.7)	12 (16.9)	0 (0.0)	43 (60.6)
7 to 90 days^†^							
Overall	137	5 (3.6)	17 (12.4)	80 (58.4)	18 (13.1)	30 (29.2)	100 (73.0)
Antihypertensive regimen	136	5 (3.7)	17 (12.5)	79 (58.1)	17 (12.5)	40 (29.4)	99 (72.8)
Diabetes regimen	71	2(2.8)	8 (11.3)	35 (49.3)	18 (25.4)	18 (25.4)	48 (67.6)

^*^Unsteadiness includes patient report of experiencing dizziness, orthostatic symptoms, syncope, falls, or feelings of unsteadiness. ^†^Adverse events in days 7 to 90 do not include those reported in first 7 days following discharge. Fourteen patients did not complete 90-day follow-up surveys, 4 died or transitioned to hospice and 10 withdrew or were lost to follow-up

## DISCUSSION

In this prospective study of older adults discharged home with cardiometabolic medication changes, we characterized the complexity of medication changes experienced by older adults in the post-hospitalization period and identified multiple gaps in discharge medication planning and follow-up. This study extends and builds on prior studies using pharmacy claims to identify medication changes made following hospitalization, as claims data cannot reliably assess medication stops and holds or patient understanding of changes.^3–5^ The observed gaps culminated in more than one-third of older adults taking changed cardiometabolic medications differently than prescribed in the week following hospitalization. Medication errors were most common among medically complex older adults prescribed five or more cardiometabolic medications. Furthermore, one-third of medication changes were subsequently modified and additional medication errors occurred in half of the participants in the 7-to-90-day post-hospital period reflecting the further challenges of medication complexity as care is transitioned back to the ambulatory setting.

Medication related errors remain a leading cause of preventable ED visits and readmissions of older adults.^8,23^ Prior studies have documented that clinical teams typically spend little time discussing medications on the day of discharge^12^ and that discharge instructions are often unclear. Thus, our finding that over one-third of older adults were taking cardiometabolic medications incorrectly following discharge is highly concerning. Furthermore, the finding that medically complex older adults using higher numbers of medications at baseline were more likely to experience errors suggests that home medication regimen complexity and polypharmacy should be a key driver in decision-making around whether to make changes to home medications on hospital discharge.

Chronic medications are commonly changed during hospitalization, yet quality improvement initiatives have largely focused on admission and discharge medications reconciliation. Hospitalizations should be viewed as a pivotal piece of a complex continuum of chronic cardiometabolic care for which greater integration is needed. Medication reconciliation is necessary but insufficient and our study findings suggest that the quality of medication discharge planning is a key area to focus future interventions. For all changes to chronic medications, patients should be educated on intended effects, side effects, home monitoring, and the need for ambulatory follow-up. While specific guidelines on home monitoring of blood pressure or blood glucose after hospitalization are limited, home monitoring is likely indicated in most patients started on new cardiometabolic medications during hospitalization to ensure both safety efficacy upon return home.^24–26^ Monitoring may be equally important when cardiometabolic medications are stopped or held which may lead to elevated blood pressure or blood glucose requiring clinical intervention. This study’s finding that one-third of discharge medication changes were further modified in the 90 days after discharge and that 17% of medication holds were not restarted further emphasizes the importance of comprehensive medication discharge planning.

Reliance only on recommending ambulatory follow-up to monitor hospital-initiated medication changes is problematic. In this study, one-quarter of patients recommended primary care or specialist follow-up did not receive it in the time advised by the inpatient team or at all. As the study required patients to have pre-existing primary care in the same health system as the hospital, these follow-up rates are higher than often seen in other settings. Patients without pre-existing primary care and patients receiving primary care at hospitals not in the same health system as their PCP face larger barriers to coordinating care.^27^ Overall, among traditional Medicare beneficiaries, fewer than half receive timely primary care follow-up after hospitalization and far fewer receive transitional care management visits.^28^

This study has limitations. Medication use relied on patient self-report which is subject to recall bias. While a study strength was the enrollment of a population with high medical complexity and frailty, as a single-center study findings may not be generalizable to other hospitals or to surgical services. Generalizability is limited by the exclusion of patients with limited English proficiency and those with cognitive impairment, both populations which may face a higher risk of medication errors. Participants may have had different medication use patterns than non-participants, who were more often older, Black, and discharged with home health services. This study focused on antihypertensive and glucose-lowering medications because these are among the most common medication classes used by older adults and modified during hospitalization, but they may be used for multiple indications and we did not assess whether changes were directly related to the primary reason for hospitalization or in response to inpatient blood pressure or glucose readings.

This prospective cohort study of older adults discharged home with cardiometabolic medication changes demonstrated the complexity of medication changes patients face following hospitalization, identified large gaps in medication discharge planning, and revealed that one-third of patients had medication errors within 7 days of discharge. Steps to ensure all patients receive high-quality medication discharge planning are urgently needed.

## Supplementary Information

Below is the link to the electronic supplementary material.

ESM1 (DOCX 206 KB)
