# Supplementary material for: Medication Errors and Gaps in Medication Discharge Planning for Hospitalized Older Adults: A Prospective Cohort Study
Source: J Gen Intern Med. 2025 Nov 19;41(3):697–706. doi: 10.1007/s11606-025-09973-x (PMC12638008; doi:10.1007/s11606-025-09973-x)

**Gaps in Care for Older Adults Discharged from the Hospital with Changes to Cardiometabolic Medications: A Prospective Cohort Study**

**Supplemental Content**

**eTable 1** Eligible Medication Classes

**eFigure 1.** Cohort Diagram

**eTable 2.** Characteristics of participants compared to eligible nonparticipants

**eTable 3.** Medication changes at hospital discharge, by medication class

**eFigure 2.** Clinic follow-up within 90 days of discharge

**eTable 1** Eligible Medication Classes

| **Antihypertensive classes** | **Glucose-lowering classes** |
| --- | --- |
| Alpha agonist | Biguanide |
| Alpha-1 blocker | Dipeptidyl peptidase-4 inhibitor |
| Angiotensin-converting enzyme inhibitor | Glucagon-like peptide-1 receptor agonist |
| Angiotensin-receptor blocker | Insulin, basal |
| Beta blocker | Insulin, prandial |
| Calcium-channel blocker | Meglitinide |
| Direct renin inhibitor | Sodium glucose transport protein 2 inhibitor |
| Loop diuretic | Sulfonylurea |
| Potassium-sparing diuretic | Thiazolidinedione |
| Thiazide diuretic |  |
| Neprilysin inhibitor |  |
| Vasodilator |  |

**eTable 2.** 7-Day Post-Discharge Survey

| **For each of the following activities, do you need help in order to complete it?*** | | | | | | | |
| --- | --- | --- | --- | --- | --- | --- | --- |
| Bathe (which include: (1) bathing more than one part of the body and (2) getting in or out of the tub or shower? | Yes | No |  |  |  |  |  |
| Dress (defined as helping oneself dress) | Yes | No |  |  |  |  |  |
| Transfer (defined as moving from bed to chair) | Yes | No |  |  |  |  |  |
| Toilet (defined as transferring to toilet, cleaning self, or using bedpan or commode) | Yes | No |  |  |  |  |  |
| Eat (defined as partial or total help feeding or requiring parenteral feeding) | Yes | No |  |  |  |  |  |
| Fatigue: “How much of the time during the past 4 weeks have you felt tired?” | All the time | Most of the time | Some of the time | A little of the time | None of the time |  |  |
| Resistance: “By yourself and not using aids, do you have any difficulty walking up 10 steps without resting?” | Yes | No |  |  |  |  |  |
| Ambulation: “By yourself and not using aids, do you have any difficulty walking several hundred yards?” | Yes | No |  |  |  |  |  |
| **For these 11 conditions, has a doctor ever told you that you have one of these conditions?** | | | | | | | |
| Hypertension | Yes | No |  |  |  |  |  |
| Diabetes | Yes | No |  |  |  |  |  |
| Cancer (other than minor skin cancer) | Yes | No |  |  |  |  |  |
| Chronic lung disease | Yes | No |  |  |  |  |  |
| Heart attack | Yes | No |  |  |  |  |  |
| Congestive heart failure | Yes | No |  |  |  |  |  |
| Angina (or chest pain) | Yes | No |  |  |  |  |  |
| Asthma | Yes | No |  |  |  |  |  |
| Arthritis | Yes | No |  |  |  |  |  |
| Stroke | Yes | No |  |  |  |  |  |
| Kidney disease | Yes | No |  |  |  |  |  |
| Next, how much do you currently weigh with your clothes on, but without your shoes? | [text box] |  |  |  |  |  |  |
| One year ago, how much do you weigh with your clothes on, but without your shoes? | [text box] |  |  |  |  |  |  |
| **Next, we will ask you about your care during your recent hospitalization at () that was from dates of: [admission date] to [discharge date]. How much would you agree with these following statements, your options are: strongly disagree, disagree, agree, and strongly agree.** | | | | | | | |
| The hospital staff took my preferences and those of my family or caregivers into account in deciding what my health care needs would be when I left the hospital. | Strongly disagree | Disagree | Agree | Strongly agree | Don’t Know/Don’t Remember |  |  |
| When I left the hospital, I had a good understanding of the things I was responsible for in managing my health. | Strongly disagree | Disagree | Agree | Strongly agree | Don’t Know/Don’t Remember |  |  |
| When I left the hospital, I clearly understood the purpose for taking each of my medications. | Strongly disagree | Disagree | Agree | Strongly agree | Don’t Know/Don’t Remember |  |  |
| **Next, we will ask you about medications which you take for high blood pressure and/or diabetes** | | | | | | | |
| Who is the primary person that manages your medications? | Myself | Family member | Nurse | Other |  |  |  |
| When you left the hospital, were you taking any medications for high blood pressure or diabetes (either given to you by the hospital team or that you took prior to being hospitalized?) | Yes | No |  |  |  |  |  |
| Additional comments/notes related to patient understanding of their medications | [text box] |  |  |  |  |  |  |
| How many medication(s) like this are you prescribed? | 1 | 2 | 3 | 4 | 5 | 6 | ≥7 |
| Repeat for each mentioned medication | What is the name of the medication? | [text box] |  |  |  |  |  |
|  | How many times a day do you take the medication? | [text box] |  |  |  |  |  |
|  | What is the dose of the medication? | [text box] |  |  |  |  |  |
|  | Was the medication changed by the hospital team? | Yes | No |  |  |  |  |
|  | [if yes] In what way was the medication changed? | Yes | No |  |  |  |  |
|  | [if yes] Why was the medication changed? | Yes | No |  |  |  |  |
|  | [if yes] Were you informed of any potential side effects from this medication? | Yes | No |  |  |  |  |
|  | [if yes] Were you instructed to measure your blood pressure or blood glucose at home? | Yes | No |  |  |  |  |
|  | [if yes] Do you have a home blood pressure/glucose monitor or other way to measure your blood pressure/sugar outside of the doctor’s office? | Yes | No |  |  |  |  |
|  | [if yes] Were you instructed to complete any follow up on your blood pressure or blood sugar after leaving the hospital? | Yes | No |  |  |  |  |
| Were there any additional blood pressure or diabetes prescriptions you were taking before being hospitalized, which you were instructed to stop taking when you left the hospital? | Yes | No |  |  |  |  |  |
| [if yes] How many medications were you instructed to stop? | 1 | 2 | 3 | 4 | ≥5 |  |  |
| Repeat for each stopped medication | What is the name of the medication? | [text box] |  |  |  |  |  |
|  | What is the dose of this medication? | [text box] |  |  |  |  |  |
|  | Was this medication changed by a physician during your stay in the hospital? | Yes | No |  |  |  |  |
|  | Why was this medication change? | [text box] |  |  |  |  |  |
|  | Were you informed of any potential side effects or harms from this medication being stopped? | Yes | No |  |  |  |  |
|  | Were you instructed to measure your blood pressure or blood glucose at home? | Yes | No |  |  |  |  |
|  | Do you have a home blood pressure/glucose monitor or other way to measure your blood pressure or blood sugar after leaving the hospital? | Yes | No |  |  |  |  |
|  | Were you instructed to complete any follow up on your blood pressure or blood sugar after leaving the hospital? | Yes | No |  |  |  |  |
| **[List of chart reviewed medications]** | | | | | | | |
| [for interviewer] Did the patient mention all the diabetes or blood pressure medications that they take? | Yes | No |  |  |  |  |  |
| [if no, for interviewer] How many diabetes and blood pressure medications have not been mentioned? | 1 | 2 | 3 | 4 | 5 |  |  |
| Repeat for each missed medication | [if no, for interviewer] What is the name of the medication? | [text box] |  |  |  |  |  |
|  | [if no, medication 1] In your medical record it appears that you were taking [medication name]. Does this sound familiar? Are you still taking it? | Yes | No |  |  |  |  |
|  | [if yes] How many times a day do you take the medication? | [text box] |  |  |  |  |  |
|  | [if yes] What is the dose of the medication? | [text box] |  |  |  |  |  |
|  | [if yes] What do you take this medication for? | [text box] |  |  |  |  |  |
|  | [if yes] Was this medication changed by the hospital team? | Yes | No |  |  |  |  |
|  | [if yes] In what way was the medication change? | Medication stopped | Medication started | Dose increased | Dose decreased |  |  |
|  | [if yes] Why was this medication changed? | [text box] |  |  |  |  |  |
|  | [if yes] were you informed of any potential effects or harms from this medication? | Yes | No |  |  |  |  |
| **Since coming home from the hospital, have there been any changed in your blood pressure/diabetes medications which you have made, either due to instructions from a healthcare provider or due to your own choice?** | Yes | No |  |  |  |  |  |
| [if yes] How many medication(s) are there that this has happened with? | 1 | 2 | 3 | ≥4 |  |  |  |
| Repeat for each medication changed post-discharge | [if yes] What is the name of this medication? | [text box] |  |  |  |  |  |
|  | [if yes] In what way was the medication changed? | Medication stopped | Medication started | Dose increased | Dose decreased |  |  |
|  | [if yes] Why was this medication changed? | [text box] |  |  |  |  |  |
|  | [if yes] Who instructed you to change this medication? | Primary care practitioner | Inpatient hospitalist | I changed it myself | Specialist | Other |  |
|  | [if specialist] What specific kind of specialist told you this? | [text box] |  |  |  |  |  |
|  | [if other] Other individual who changed this medication? | [text box] |  |  |  |  |  |
| **Next, we will ask you about your health since leaving the hospital. Since leaving the hospital, have you experienced any of the symptoms of:** | | | | | | | |
| Dizziness, lightheadedness, feeling faint, or feeling like you might black out? | Yes | No |  |  |  |  |  |
| Lightheadedness or dizziness when you move from lying to standing (for example: getting out of bed) | Yes | No |  |  |  |  |  |
| Passing out, fainting, losing consciousness | Yes | No |  |  |  |  |  |
| Falls (with or without injury from falling) | Yes | No |  |  |  |  |  |
| Worries about falling or feels of unsteadiness when standing or walking? | Yes | No |  |  |  |  |  |
| Low blood sugar (hypoglycemia) episodes that you felt? | Yes | No |  |  |  |  |  |
| Low blood sugar (hypoglycemia) episodes that you did not feel? | Yes | No |  |  |  |  |  |
| Since leaving the hospital, have you been to the emergency department or been admitted to the hospital? | Yes | No |  |  |  |  |  |
| [if yes] What was the primary reason for your visit? | [text box] |  |  |  |  |  |  |
| [if yes] What hospital did you visit? | [text box] |  |  |  |  |  |  |

*Of the Katz ADL Index, the question on independence of continence is missing

**eTable 3.** 90-Day Post-Discharge Survey

| **Medications** | | | | | | | |
| --- | --- | --- | --- | --- | --- | --- | --- |
| Are you currently taking any medications for high blood pressure or diabetes? | Yes | No |  |  |  |  |  |
| How many of these medication(s) are you taking? | 1 | 2 | 3 | 4 | 5 | 6 | ≥7 |
| Repeat for each mentioned medication | What is the name of the medication? | [text box] |  |  |  |  |  |
|  | Is this a diabetes or antihypertensive medication? | Diabetes | Antihypertensive |  |  |  |  |
|  | How many times a day do you take the medication? | [text box] |  |  |  |  |  |
|  | What is the dose of the medication? | [text box] |  |  |  |  |  |
| Since our last survey, have there been any changes to your blood pressure/diabetes medication(s), either due to instruction from healthcare provider or due to your own choice? | Healthcare provider | Own choice | No |  |  |  |  |
| How many of these medications have been changed? | 1 | 2 | 3 | 4 | More |  |  |
| Repeat for each mentioned medication | What is the name of the medication? | [text box] |  |  |  |  |  |
|  | What is the dose of this medication? | [text box] |  |  |  |  |  |
|  | How many times a day do you take this medication? | [text box] |  |  |  |  |  |
|  | In what way was the medication changed? | Medication stopped | Medication started | Dose increase | Dose decrease |  |  |
|  | Why was the medication changed | [text box] |  |  |  |  |  |
|  | Who instructed you to change this medication? | Primary care provider | Specialist | Changed it myself | Other |  |  |
| **[List of chart reviewed medications]** | | | | | | | |
| [for interviewer] Did the patient mention all the diabetes or blood pressure medications that they take? | Yes | No |  |  |  |  |  |
| [if no, for interviewer] How many diabetes and blood pressure medications have not been mentioned? | 1 | 2 | 3 | 4 | 5 | 6 |  |
| Repeat for each missed medication | [if no, for interviewer] What is the name of the medication? | [text box] |  |  |  |  |  |
|  | [if no, medication 1] In your medical record it appears that you were taking [medication name]. Are you still taking it? | Yes | No |  |  |  |  |
|  | [if yes] How many times a day do you take the medication? | [text box] |  |  |  |  |  |
|  | [if yes] What is the dose of the medication? | [text box] |  |  |  |  |  |
|  | [if yes] What do you take this medication for? | [text box] |  |  |  |  |  |
|  | [if yes] Was this medication changed? In what way was it changed? | No change | Medication stopped | Medication stared | Dose increased | Dose decreased |  |
|  | [if changed] Why was this medication changed? | [text box] |  |  |  |  |  |
|  | [if changed] were you informed of any potential effects or harms from this medication? | Yes | No |  |  |  |  |
| **Next, we will ask you about your health since leaving the hospital. Since leaving the hospital, have you experienced any of the symptoms of:** | | | | | | | |
| Dizziness, lightheadedness, feeling faint, or feeling like you might black out? | Yes | No |  |  |  |  |  |
| Lightheadedness or dizziness when you move from lying to standing (for example: getting out of bed) | Yes | No |  |  |  |  |  |
| Passing out, fainting, losing consciousness | Yes | No |  |  |  |  |  |
| Falls (with or without injury from falling) | Yes | No |  |  |  |  |  |
| Worries about falling or feels of unsteadiness when standing or walking? | Yes | No |  |  |  |  |  |
| Low blood sugar (hypoglycemia) episodes that you felt? | Yes | No |  |  |  |  |  |
| Low blood sugar (hypoglycemia) episodes that you did not feel? | Yes | No |  |  |  |  |  |
| In the past 3 months since our last survey, have you been to the emergency department or been admitted to the hospital? | Yes | No |  |  |  |  |  |
| [if yes] How many times has this happened? | 1 | 2 | 3 | 4 |  |  |  |

**eFigure 1.** Cohort Diagram

Hospital discharges to home of older adults on medicine, cardiology, or neurology services (n = 2,290)

Discharges with cardiometabolic medication changes (n = 1,039)

No cardiometabolic medications (n = 328)

No cardiometabolic medication changes (n = 924)

Re-hospitalized or reported to be in altered mental state at time of contact (n = 60)

Previously contacted about study during prior hospitalization (n = 119)

Unable to reach (n = 203)

Contacted (n = 657)

Enrolled (n = 168)

Found to be ineligible after contact* (n = 57)

Declined participation (n = 432)

7-day survey completion

(n = 151)

Withdrew or did not complete 1^st^ survey (n = 17)

90-day survey completion

(n = 137)

Hospice care or passed away (n = 4)

Withdrawn (n = 5)

Lost to follow up (n = 5)

***** Reasons for ineligibility after phone contact include self-report of not managing medications, primary language other than English, and inability to pass Blessed Short Test (Short Orientation-Memory-Concentration Test).

**eTable 4.** Characteristics of participants compared to eligible nonparticipants

| **Characteristic, No. (%)** | | **Participants** | **Eligible non-participants** | **Standardized mean difference** |
| --- | --- | --- | --- | --- |
| **No.** | | 151 | 888 |  |
| **Demographics** | |  |  |  |
| **Age, years, median (IQR)** | | 74 (70-78) | 77 (71-83) | **0.49** |
| **Male** | | 82 (54.3) | 430 (48.4) | **0.11** |
| **Race** | |  |  | **0.40** |
|  | White | 124 (82.1) | 604 (68.0) |  |
|  | Black | 26 (17.2) | 201 (22.6) |  |
|  | Asian | 0 (0.0) | 18 (2.0) |  |
|  | American Indian/Alaska Native | 0 (0.0) | 3 (0.3) |  |
|  | Other | 0 (0.0) | 51 (5.7) |  |
|  | Unknown | 1 (0.7) | 11 (1.2) |  |
| **Ethnicity** | |  |  | 0.09 |
|  | Hispanic or Latino | 2 (1.3) | 22 (2.5) |  |
|  | Not Hispanic or Latino | 146 (96.7) | 840 (94.6) |  |
|  | Unknown | 3 (2.0) | 26 (2.9) |  |
| **Hospitalization Characteristics** | |  |  |  |
| **Discharge disposition** | |  |  | **0.51** |
|  | Home | 109 (72.2) | 427 (48.1) |  |
|  | Home with services | 42 (27.8) | 461 (51.9) |  |
| **Discharge service** | |  |  | **0.23** |
|  | Cardiology | 84 (55.6) | 397 (44.7) |  |
|  | Medicine | 65 (43.0) | 469 (52.8) |  |
|  | Neurology | 2 (1.3) | 22 (2.5) |  |
| **Length of stay, days** | | 3 (2-6) | 4.5 (3-8) | **0.26** |
| **Primary care group practice** | |  |  | 0.05 |
|  | Academic | 62 (41.4) | 339 (38.2) |  |
|  | Community | 82 (54.3) | 508 (57.2) |  |
|  | Community health center | 7 (46.4) | 41 (4.6) |  |
| **Admission Medication Use** | |  |  |  |
| **Any antihypertensive medication** | | 140 (92.7) | 811 (91.3) | 0.09 |
| **Any glucose-lowering medication** | | 67 (44.4) | 325 (36.6) | 0.07 |
| **Median (IQR) No. cardiometabolic medications** | | 3 (2-4) | 3 (2-4) | 0.02 |
| **Antihypertensive medication classes** | |  |  |  |
|  | Angiotensin-converting enzyme inhibitor | 43 (28.5) | 196 (22.1) | **0.15** |
|  | Alpha agonist | 1 (0.7) | 10 (1.1) | 0.05 |
|  | Alpha-1 blocker (approved for hypertension) | 1 (0.7) | 9 (1.0) | 0.04 |
|  | Angiotensin II receptor blocker | 46 (30.5) | 261 (29.4) | 0.02 |
|  | Beta blocker | 80 (53.0) | 506 (57.0) | 0.08 |
|  | Calcium channel blocker | 48 (31.8) | 289 (32.5) | 0.02 |
|  | Direct renin inhibitor | 0 (0.0) | 1 (0.1) | 0.5 |
|  | Loop diuretic | 46 (30.5) | 391 (44.0) | **0.31** |
|  | Neprilysin inhibitor | 2 (1.3) | 42 (4.7) | **0.20** |
|  | Potassium sparing diuretic | 20 (13.2) | 146 (16.4) | 0.09 |
|  | Thiazide diuretic | 37 (24.5) | 142 (16.0) | **0.21** |
|  | Vasodilator | 17 (11.3) | 92 (10.4) | 0.03 |
| **Glucose-lowering medication classes** | |  |  |  |
|  | Basal insulin | 26 (17.2) | 114 (12.8) | **0.12** |
|  | Biguanide | 35 (23.2) | 147 (16.6) | **0.17** |
|  | Dipeptidyl peptidase-4 inhibitor | 5 (3.3) | 33 (3.7) | 0.02 |
|  | Glucagon-like peptide-1 receptor agonist | 13 (8.6) | 69 (7.8) | 0.03 |
|  | Meglitinide | 1 (0.7) | 5 (0.6) | 0.01 |
|  | Prandial insulin | 12 (8.0) | 53 (6.0) | 0.08 |
|  | Sodium-glucose transport protein-2 inhibitor | 13 (8.6) | 109 (12.3) | **0.12** |
|  | Sulfonylurea | 7 (4.6) | 57 (6.4) | 0.08 |
|  | Thiazolidinedione | 0 (0.0) | 1 (0.1) | 0.05 |

**Note:** Bolded values of standardized mean differences >0.1, a common threshold for meaningful difference.

**eTable 5.** Medication changes at hospital discharge, by medication class

| **Class** | **All changes** | **Starts** | **Stops** | **Holds** | **Dose increases** | **Dose decreases** |
| --- | --- | --- | --- | --- | --- | --- |
| **Overall*** | 319 | 108 | 67 | 73 | 36 | 33 |
| **Antihypertensive medication classes** | | | | | |  |
| **Overall** | 285 | 95 | 62 | 66 | 31 | 30 |
| **ACE-I** | 30 | 5 | 9 | 12 | 3 | 1 |
| **Alpha agonist** | 1 | 1 | 0 | 0 | 0 | 0 |
| **ARB** | 38 | 12 | 7 | 13 | 1 | 4 |
| **Beta blocker** | 75 | 31 | 14 | 9 | 10 | 11 |
| **Calcium channel blocker** | 34 | 11 | 8 | 7 | 5 | 3 |
| **Loop diuretic** | 41 | 16 | 6 | 5 | 7 | 7 |
| **Neprilysin inhibitor** | 5 | 3 | 1 | 0 | 0 | 1 |
| **Potassium-sparing diuretic** | 17 | 12 | 0 | 2 | 2 | 1 |
| **Thiazide diuretic** | 30 | 0 | 13 | 16 | 1 | 0 |
| **Vasodilator** | 14 | 4 | 4 | 2 | 2 | 2 |
| **Glucose-lowering medication classes** | | | | | |  |
| **Overall** | 34 | 13 | 5 | 7 | 5 | 3 |
| **Biguanide** | 7 | 0 | 2 | 3 | 1 | 1 |
| **DPP4** | 2 | 1 | 1 | 0 | 0 | 0 |
| **Insulin, basal** | 10 | 3 | 0 | 1 | 3 | 2 |
| **Insulin, prandial** | 4 | 2 | 0 | 1 | 1 | 0 |
| **SGLT-2** | 10 | 6 | 2 | 2 | 0 | 0 |
| **Sulfonylurea** | 1 | 1 | 0 | 0 | 0 | 0 |

**Abbreviations**: ACE-I, angiotensin-converting enzyme inhibitor; ARB, angiotensin II receptor blocker, DPP4, dipeptidyl peptidase-4 inhibitor, SGLT-2, Sodium-glucose transport protein-2 inhibitor

**Note**: No alpha-1 blockers, glucagon-like peptide-1 receptor agonists, or meglitinides were changed at hospital discharge.

*An additional two medications (one angiotensin II receptor blocker and one basal insulin) had changes in frequency but not dose.

**eTable 6.** Factors associated with medication errors between 7 and 90 days post-discharge

| **Characteristic** | **No. with error / Total No. (%)** | **Incident rate ratio (95% CI)** | **Adjusted incident rate ratio (95% CI)** |
| --- | --- | --- | --- |
| **Overall** | 69/137 (50.4) | n/a | n/a |
| **Demographics** |  |  |  |
| **Age** |  |  |  |
| <75 | 42/79 (53.2) | Ref | - |
| ≥75 | 27/58 (46.6) | 0.88 (0.62 to 1.24) | - |
| **Sex** |  |  |  |
| Female | 25/62 (40.3) | Ref | Ref |
| Male | 44/75 (58.7) | **1.45 (1.02 to 2.08)** | 1.28 (0.91 to 1.80) |
| **Race** |  |  |  |
| White | 54/113 (47.8) | Ref | - |
| Non-white | 15/24 (62.5) | 1.31 (0.91 to 1.88) | - |
| **Hospitalization characteristics** |  |  |  |
| **Discharge disposition** |  |  |  |
| Home | 50/101 (49.5) | Ref | - |
| Home with services | 19/36 (52.8) | 1.07 (0.74 to 1.54) | - |
| **Discharge service** |  |  |  |
| Medicine | 30/56 (53.6) | Ref | - |
| Cardiology/neurology | 39/81 (48.2) | 0.90 (0.64 to 1.25) | - |
| **Length of stay, days** | 69/137 (50.4) | 1.02 (0.98 to 1.05) | - |
| **Primary care practice** |  |  |  |
| Academic | 30/55 (54.6) | Ref | - |
| Community/Community health center | 39/82 (47.6) | 1.15 (0.82 to 1.60) | - |
| **No. of cardiometabolic medications** |  |  |  |
| < 5 | 30/86 (34.9) | Ref | Ref |
| > 5 | 39/51 (76.5) | **2.19 (1.58 to 3.04)** | **1.66 (1.13 to 2.45)** |
| **No. of cardiometabolic medication changes** |  |  |  |
| 1 | 10/33 (30.3) | Ref | Ref |
| > 2 | 59/104 (56.7) | **1.87 (1.09 to 3.23)** | 1.42 (0.82 to 2.46) |
| **Cardiometabolic medication classes** |  |  |  |
| Single | 26/71 (36.6) | Ref | Ref |
| Both | 43/66 (65.2) | **1.78 (1.25 to 2.53)** | 1.27 (0.88 to 1.85) |
| **Primary care follow-up** |  |  |  |
| Completed | 65/126 (51.6) | Ref | - |
| Not completed | 4/11 (36.4) | 1.42 (0.64 to 3.16) | - |
| **Specialist care follow-up** |  |  |  |
| Completed | 49/99 (49.5) | Ref | - |
| Not completed | 20/38 (52.6) | 0.94 (0.66 to 1.35) | - |
| Clearly understood purpose of medications at discharge * |  |  |  |
| Strongly agree/Agree | 62/127 (48.8) | Ref | - |
| Strongly disagree/Disagree | 7/10 (70.0) | 1.43 (0.92 to 2.23) | - |
| **Self-reported health status** |  |  |  |
| **FRAIL Scale** |  |  |  |
| Not frail | 39/81 (48.1) | Ref | - |
| Frail | 30/56 (53.6) | 1.11 (0.80 to 1.55) | - |
| **Activities of Daily Living** |  |  |  |
| No assistance needed | 51/107 (47.7) | Ref | Ref |
| Any assistance needed | 18/30 (60.0) | 1.26 (0.88 to 1.79) | 1.11 (0.81 to 1.53) |
| **1-Year Risk of Mortality** |  |  |  |
| Moderate | 28/66 (42.4) | Ref | - |
| High | 41/71 (57.7) | 1.38 (0.94 to 2.02) | - |

**Note**: Race and ethnicity measures were not included due to small sample size (see Table A). Bolded estimates are statistically significant at alpha = 0.05.

***** Assessed from question 3 of the 3-Item Care Transitions Measure “When I left the hospital, I clearly understood the purpose for taking each of my medications. “

**eFigure 2.** Clinic follow-up within 90 days of discharge

A) Primary Care Follow-Up


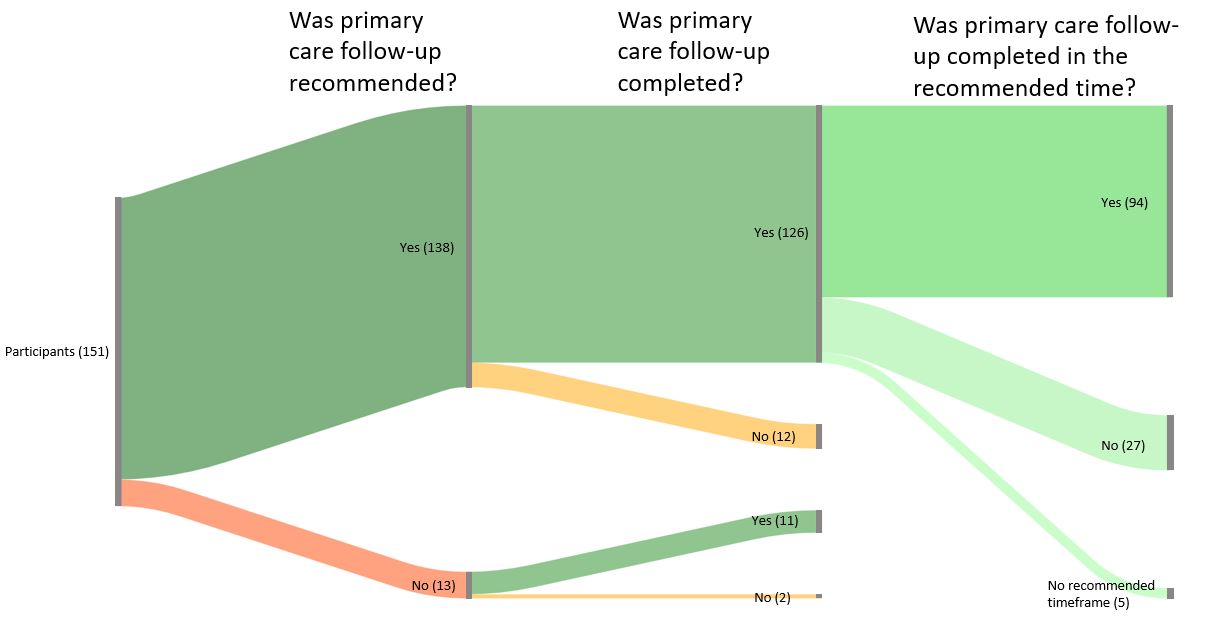


B) Specialist Follow-Up


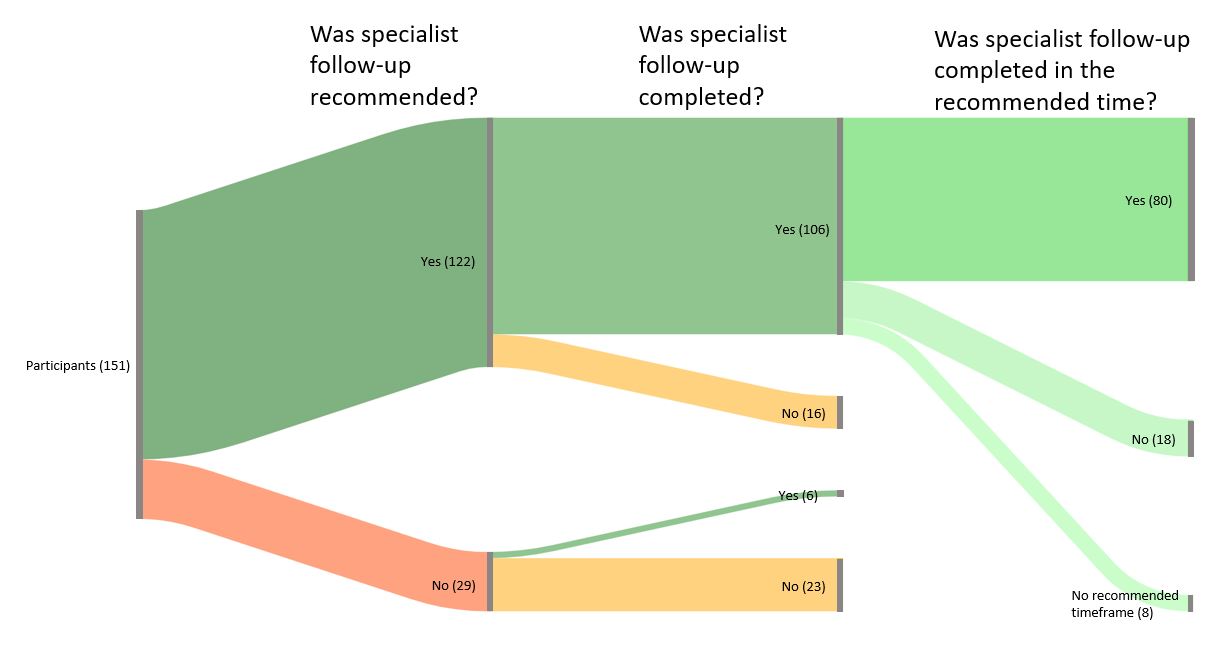

Supplement: Supplementary file 1 — (DOCX 206 KB) [file 11606_2025_9973_MOESM1_ESM.docx]
